# Daily physical activity volume, intensity, and accumulation patterns in relation to all-cause mortality in the German National Cohort (NAKO)

**DOI:** 10.1186/s12916-026-05385-8

**Published:** 2026-11-10

**Authors:** Patricia Fromherz, Michael J. Stein, Claas Lendt, Fabian Bamberg, Hansjörg Baurecht, Mirko Brandes, Julian Brummer, Beate Fischer, Florian Herbolsheimer, Thomas Keil, Lilian Krist, Wolfgang Lieb, Rafael Mikolajczyk, Manuela Neuenschwander, Nadia Obi, Steffen Ringhof, Börge Schmidt, Matthias B. Schulze, Virginie Stanislas, Karen Steindorf, Paula Stürmer, Antje Ullrich, Andrea Werdecker, Ronny Westerman, Michael F. Leitzmann

**Affiliations:** 1 Department of Epidemiology and Preventive Medicine, University of Regensburg, Franz- Josef-Strauss Allee 11, 93053 Regensburg, Germany; 2 Department of Neurology, University Hospital of Regensburg at medbo KU, Universitätsstraße 84, 93053 Regensburg, Germany; 3 Institute of Epidemiology, Helmholtz Zentrum München, German Research Center for Environmental Health (GmbH), Ingolstädter Landstraße 1, 85764 Neuherberg, Germany; 4 Institute for Movement Therapy and Movement-oriented Prevention and Rehabilitation, German Sport University Cologne, Cologne, Germany; 5 Department of Diagnostic and Interventional Radiology, Medical Center, Faculty of Medicine, University of Freiburg, University of Freiburg, Hugstetter Str. 55, 79106 Freiburg, Germany; 6 Department of Epidemiology and Preventive Medicine, Medical Sociology, University of Regensburg, Franz-Josef-Strauss Allee 11, 93053 Regensburg, Germany; 7 Department of Prevention and Evaluation, Leibniz Institute for Prevention Research and Epidemiology - BIPS, Achterstraße 30, 28359 Bremen, Germany; 8 Division of Physical Activity, Cancer Prevention and Survivorship, German Cancer Research Center (DKFZ), Im Neuenheimer Feld 581, 69120 Heidelberg, Germany; 9 Medical Faculty, Heidelberg University, Im Neuenheimer Feld 672, 69120 Heidelberg, Germany; 10 Institute of Social Medicine, Epidemiology and Health Economics, Charité - Universitätsmedizin Berlin, Luisenstr. 57, 10117 Berlin, Germany; 11 Institute of Clinical Epidemiology and Biometry, University of Würzburg, Am Schwarzenberg 15, Haus A15, 97078 Würzburg, Germany; 12 State Institute of Health I, Bavarian Health and Food Safety Authority, Eggenreuther Weg 43, 91058 Erlangen, Germany; 13 Institute of Epidemiology, Kiel University, Arnold Heller Straße 3 (Haus U25), 24105 Kiel, Germany; 14 Institute for Medical Epidemiology, Biometrics, and Informatics (IMEBI), Medical Faculty of the Martin, Luther University Halle-Wittenberg, Magdeburger Straße 8, 06112 Halle (Saale), Germany; 15 Interdisciplinary Center for Health Sciences, Magdeburgerstr. 8, 06114 Halle (Saale), Germany; 16 Institute for Biometrics and Epidemiology, German Diabetes Center, Leibniz Center for Diabetes Research at Heinrich Heine University Düsseldorf, Auf’m Hennekamp 65, 40225 Düsseldorf, Germany; 17 German Center for Diabetes Research, Neuherberg, Germany; 18 Institute for Occupational and Maritime Medicine (ZfAM), University Medical Center Hamburg-Eppendorf, Maritnistr. 52, Hamburg, 20246 Germany; 19 Institute for Medical Informatics, Biometry and Epidemiology, University Hospital Essen, Hufelandstraße 55, 45147 Essen, Germany; 20 Department of Molecular Epidemiology, German Institute of Human Nutrition Potsdam- Rehbruecke, Arthur-Scheunert-Allee 114-116, 14558 Nuthetal, Germany; 21 Institute of Nutritional Science, University of Potsdam, Karl-Liebknecht-Str. 24-25, 14476 Potsdam, Germany; 22 Institute of Epidemiology and Prevention, Faculty of Medicine and Medical Center, University of Freiburg, Elsässer Str. 2, 79110 Freiburg, Germany; 23 Institute for Community Medicine, Department SHIP, KEF - University Medicine Greifswald, Walther-Rathenau-Str. 48, 17475 Greifswald, Germany; 24 partner site Greifswald, German Centre for Cardiovascular Research (DZHK), University Medicine Greifswald, Fleischmannstraße 8, 17475 Greifswald, Germany; 25 Federal Institute for Population Research (BiB), Friedrich-Ebert-Allee 4, 65185 Wiesbaden, Germany

**Keywords:** Physical activity patterns, Mortality, Accelerometry, NAKO

## Abstract

**Background:**

Greater physical activity volume is associated with lower mortality, but how activity intensity and its accumulation throughout the day relate to mortality beyond volume remains unclear.

**Methods:**

We prospectively analyzed hip-worn accelerometer-derived physical activity in 65,028 participants aged 19–74 years from the German National Cohort (NAKO). Using multivariable-adjusted Cox models, hazard ratios (HR) and 95% confidence intervals (CI) for all-cause mortality were estimated. Models of daily intensity and daily accumulation (most active 1–120 min of the day, sporadically distributed or within continuous bouts) were additionally adjusted for total activity volume.

**Results:**

During a median follow-up of 8.0 years, 1,634 deaths occurred. Physical activity volume showed a linear inverse association with mortality (HR comparing 75th vs. 25th percentile = 0.73 [95% CI = 0.67–0.80]). Independent of activity volume, higher activity intensity during the most active minutes of the day was inversely associated with mortality, with stronger associations at shorter than at longer durations (e.g., HR = 0.58 [95% CI = 0.53–0.63] for the most active 1 min vs. HR = 0.69 [95% CI = 0.65–0.74] for 120 min). Associations were attenuated when these minutes were accumulated in continuous bouts (e.g., HR = 0.71 [95% CI = 0.66–0.77] for 1 min vs. HR = 0.83 [95% CI = 0.76–0.91] for 120 min). In analyses stratified by overall activity volume, higher activity intensity within continuous bouts was associated with lower mortality risk at low activity volume, whereas associations at high activity volume were absent at shorter but inverse at longer bout durations.

**Conclusions:**

Beyond total activity volume, intensity patterns of activity accumulation were associated with mortality risk. Inverse associations were strongest at shorter durations and for intermittent rather than continuous accumulation. Higher activity intensity was more strongly associated with lower mortality among individuals with low activity volume, particularly at shorter bout durations. Together, these findings support a multidimensional view of physical activity in which volume, intensity, and accumulation pattern provide complementary information for mortality risk.

**Supplementary Information:**

The online version contains supplementary material available at https://doi.org/10.1186/s12916-026-05385-8.

## Background

Insufficient physical activity represents a global public health concern and an important risk factor for premature death [1]. Most evidence on this association comes from studies using self-reported physical activity, which is prone to social desirability and recall bias and cannot accurately quantify short or sporadic bouts of physical activity. In recent years, accelerometer-based measures have greatly improved the ability to obtain more valid assessments of physical activity in large-scale population studies, enabling a more detailed evaluation of the amount, intensity, and temporal distribution of physical activity [2].

Despite these advances, important questions remain about how different indicators of physical activity relate to mortality. Specifically, the relative contributions of physical activity volume (e.g., total duration of activity) and intensity (e.g., metabolic equivalent of task [MET]) remain uncertain, with some studies identifying volume [3, 4] and others intensity [5–8] as the main driver of risk reduction. The role of physical activity accumulation patterns is also unclear. Emerging evidence suggests that the impact of physical activity intensity on mortality depends on how that activity is accumulated throughout the day. For example, moderate-intensity activity accumulated in continuous bouts has been associated with lower risk of mortality, compared to the same moderate dose accumulated sporadically across the day, whereas vigorous activity may confer greater benefit when performed in sporadic bouts, compared to the same dose of continuous vigorous activity [9]. However, most previous studies have focused on activity volume and intensity, often using cut-point–based metrics [3, 4, 8], although one study applied cut-point–free approaches to quantify these dimensions [5]. Fewer have investigated how activity accumulation patterns influence mortality risk [6, 7, 9]. Although recent studies have examined accelerometer-derived intensity metrics and activity accumulation patterns in relation to mortality, to our knowledge, no study has jointly investigated activity volume, cut-point–free intensity distribution, and activity accumulation patterns within the same analytical framework. Therefore, how these physical activity dimensions jointly relate to mortality remains insufficiently understood.

To address these gaps, we used accelerometer data from the German National Cohort (NAKO) to examine how complementary indicators of physical activity, including total activity amount (i.e., volume and duration), intensity (i.e., intensity gradient and M-metrics representing the intensity of the most active minutes of the day), and accumulation pattern (i.e., continuous bouts and bout distribution), relate to risk of all-cause mortality.

## Methods

### Study population

NAKO is a population-based prospective cohort study that enrolled 205,415 men and women aged 19 to 74 years at 18 study centers representing urban, industrial, and rural regions of Germany. At baseline between 2014 and 2019, participants completed self-administered touchscreen questionnaires and interviews, underwent physical and functional examinations, and provided biosamples. Detailed information on the study design has been published previously [10]. In brief, participants were randomly selected using an age- and sex-stratified sampling strategy, with 10,000 individuals recruited per 10-year age group between 20 and 39 years, and 26,667 per group between 40 and 69 years. The baseline response proportion was 15.6% [11]. Ethics approval was granted by the relevant local committees, and all participants provided written informed consent.

Accelerometer-based physical activity was assessed in 72,430 participants over seven consecutive days. Valid accelerometer data were defined as a minimum of two weekdays and one weekend day with at least 16 h of recorded activity per day [12]. After excluding 7,397 participants with invalid accelerometer data (1,722 during initial preprocessing and 5,675 during study-specific preprocessing) and 5 participants with missing mortality data, 65,028 individuals remained for analysis (Additional file 1: Supplementary Fig. 1).

### Physical activity data

Physical activity was measured using triaxial ActiGraph accelerometers (GT3X+, wGT3X+, and wGT3X-BT; ActiGraph, Pensacola, FL, USA) worn on the right hip. Participants were instructed to wear the device continuously for seven consecutive days starting the day after their baseline study visit, removing it only for water-based activities exceeding 30 min. Raw acceleration data were recorded at a sampling frequency of 100 Hz with the Idle Sleep Mode disabled. Raw data were processed using GGIR (version 3.2-9) in R (version 4.3.1) [13] on a high-performance computing cluster, including auto-calibration against local gravity, non-wear detection, and feature extraction [14, 15].

Six accelerometer-based metrics were derived to capture complementary indicators of daily physical activity, including overall activity amount (volume and duration), intensity (intensity gradient and M-metrics representing the intensity of the most active minutes irrespective of accumulation pattern [16]), and temporal structure of accumulation (M-continuous-metrics capturing sustained bouts and M-ratio reflecting whether activity was achieved more sporadically or continuously throughout the day) (details are provided in Additional file 1: Supplementary Table 1 [17–19]).

These were defined as follows:

1. Volume: The overall magnitude of movement across the 24-hour period (averaged across the full measurement period), calculated as average acceleration using Euclidean Norm Minus One (ENMO) and expressed in milli-gravity units (mg);
2. Duration: The total number of minutes per day of non-sedentary [20], non-sleep movement [21] across the 24-hour period;
3. Intensity gradient: The distribution of activity time across light, moderate, and vigorous intensity levels, derived by modelling time spent at successively higher acceleration levels in relation to movement intensity; higher (less negative) values indicate a greater share of high-intensity activity;
4. Mx-metrics (M1 to M120): The average acceleration during the most active minutes of the day (one to 120 minutes), irrespective of whether those minutes occurred in one continuous bout or were dispersed throughout the day;
5. Mx-continuous-metrics (M1-continuous to M120-continuous): The average acceleration during the most active continuous bouts (one to 120-minute bouts), calculated as the 25th percentile of accelerations within that bout to ensure that at least 75% of the time was spent above this intensity level while allowing for brief interruptions (e.g., waiting at traffic lights);
6. Mx-ratio: The ratio of continuous to sporadic activity intensity within the most active one to 120 minutes, where higher values indicate more continuous accumulation.

To mitigate the influence of outliers, all physical activity metrics were winsorized at the 99.9th percentile, with the exception of the intensity gradient, which was winsorized at the 0.01st percentile. Mx-ratio values were capped at 1.0 to maintain logical consistency.

### Cohort follow-up and mortality ascertainment

We used all-cause mortality as an objective endpoint that captures the overall associations of physical activity volume, intensity, and accumulation patterns with risk of death. Vital status was ascertained through active and passive follow-up, involving the collection and verification of death certificates to determine date and cause of death. Follow-up information was obtained from regular vital status surveys and secondary sources, including registry inquiries, health authorities, and other relevant documentation [22].

Vital status was ascertained through 30 April 2024 (end of the first follow-up examination) and updated with additional reported deaths through December 2025. Follow-up time was calculated from the start of accelerometry to death or censoring at the last known vital status.

### Covariates

Potential confounding variables were identified using directed acyclic graphs, informed by prior evidence [23] (Additional file 1: Supplementary Fig. 2). We adjusted for sex (women, men), age group (5-year categories), study center region (south-east, south-west, north-east, north-west, west, central Germany, and Berlin), socioeconomic status (International Socioeconomic Index of Occupational Status, continuous), employment status (employed, unemployed), alcohol consumption exceeding recommended thresholds (Alcohol Use Disorder Identification Test-Consumption [AUDIT-C] score ≥ 4 for men, ≥ 3 for women [24]), smoking status (never, former, current), self-reported prevalent physician diagnosis of cardiovascular disease and diabetes (no, yes), body mass index (kg/m^**2**^, continuous), maximum handgrip strength (kg, continuous), device-based sedentary time (hours per day, continuous), and device-based sleep duration (hours per night, continuous). Further details are provided in Additional file 1: Supplementary Table 2.

### Statistical analysis

Participant characteristics were summarized using age-standardised means for continuous variables and percentages for categorical variables. Spearman rank correlation coefficients were used to examine correlations among physical activity metrics. Missing covariate data were addressed using multiple imputation by chained equations (ten imputed datasets with five iterations each). Predictive mean matching was used for continuous variables, logistic regression for binary variables, and polytomous logistic regression for nominal variables. Convergence and plausibility of the imputations were assessed visually [25].

Multivariable-adjusted Cox proportional hazards regression models, with age as the underlying time scale, were used to estimate hazard ratios (HRs) and 95% confidence intervals (CIs) for the relations of physical activity amount (volume and duration), intensity (intensity gradient and Mx-metrics), and temporal structure (Mx-continuous-metrics and accumulation pattern) to all-cause mortality. All exposures were modeled as continuous variables. Linearity was assessed using restricted cubic splines with knots placed at the 10th, 50th, and 90th percentiles; when the linearity assumption was satisfied, exposures were modeled as linear terms. For interpretability, HRs comparing the 50th and 75th percentiles with the 25th percentile were estimated. The proportional hazards assumption was assessed using Schoenfeld residuals and visual inspection. Multicollinearity was assessed using variance inflation factors (VIFs), calculated separately for each imputed dataset and summarized across imputations.

Models examining intensity gradient and temporal structure were adjusted for total physical activity volume. This allowed for the estimation of associations independent of overall physical activity, capturing complementary indicators of the 24-hour activity profile [16]. To investigate whether the association between continuous-bout physical activity intensity and mortality was modified by physical activity volume, multiplicative interaction terms were tested, and analyses stratified by activity volume were conducted.

Interactions between physical activity amount, intensity gradient, and all covariates were also examined to identify potential effect modification across subgroups. Interaction tests were conducted by including cross-product terms in the model and evaluating the corresponding coefficients using Wald tests, with false discovery rate adjustment for multiple testing. All P-values were derived from two-sided tests, with statistical significance defined as α = 0.05.

To assess the robustness of our findings, numerous sensitivity analyses were conducted. First, the first two years of follow-up were excluded to address potential reverse causation. Second, participants with prevalent cancer except non-melanoma skin cancer were excluded. Third, models were re-estimated after removing terms for prevalent cardiovascular disease, diabetes, and body mass index from the adjustment set, as these factors may represent mediators rather than confounders of the physical activity-mortality association.

All statistical analyses were performed using R version 4.2.3; accelerometry processing on the high-performance computing cluster was performed using R version 4.3.1 [13]. Multiple imputation was performed using the *mice* package [25] and Cox regression models were fitted using the *rms* package [26].

## Results

We first describe baseline characteristics and interrelationships among the physical activity metrics, followed by analyses of their associations with risk of all-cause mortality, progressing from overall activity amount to intensity and temporal structure.

### Baseline characteristics by total physical activity volume and intensity gradient

We examined age-standardised baseline characteristics descriptively to identify potential confounding factors. Among 65,028 participants (mean age 50.0 years; 52.1% women), those in the lowest quartile of total physical activity volume had a mean daily acceleration of 7.8 m*g*, whereas those in the highest quartile had a mean daily acceleration of 16.5 m*g*, more than twice as high. Correspondingly, average activity duration was 242.7 min and 406.6 min per day in the lowest and highest quartiles of activity volume, respectively, while sedentary time was markedly higher in the lowest quartile compared to the highest (7.8 vs. 5.9 h/day). High vs. low total physical activity volume was characterized by more favourable health characteristics, including a lower proportion of current smoking, lower mean body mass index, lower proportion of cardiovascular disease and diabetes, and a greater proportion of employed participants. However, higher activity volume was characterized by higher proportion of participants reporting high alcohol consumption above recommended thresholds. Socioeconomic status, grip strength, and sleep duration were broadly similar across extreme quartiles of activity volume.

Physical activity intensity gradient values ranged from − 3.1 in the lowest quartile to − 2.4 in the highest quartile (less negative values indicating a greater share of high-intensity activity). The distribution of potential confounding variables across intensity gradient quartiles was largely similar to that across activity volume, except that participants in extreme intensity gradient quartiles accumulated comparable daily activity minutes, while socioeconomic status was higher among those with greater intensity gradient (Table 1).

**Table 1:** Baseline characteristics of participants in the German National Cohort by quartiles of physical activity volume and intensity gradient (Q1, Q4)

Variable	Physical activity volume				Physical activity intensity gradient				Full cohort
	Q1 (lowest volume)	Q2	Q3	Q4 (highest volume)	Q1 (lowest intensity gradient)	Q2	Q3	Q4 (highest intensity gradient)	
**N**	16,296	16,274	16,238	16,220	16,296	16,274	16,238	16,220	65,028
**Age (years)**	50.0 ± 12.6	50.0 ± 12.6	50.0 ± 12.6	50.0 ± 12.6	50.0 ± 12.6	50.0 ± 12.6	50.0 ± 12.6	50.0 ± 12.6	50.0 ± 12.6
**Physical activity volume (ENMO**^1^, **m*g*)**	7.8 ± 1.4	10.3 ± 1.1	12.3 ± 1.2	16.5 ± 3.3	9.6 ± 2.9	11.1 ± 2.9	12.0 ± 3.1	14.2 ± 4.2	11.7 ± 3.7
**Physical activity intensity gradient**	-2.9 ± 0.3	-2.8 ± 0.2	-2.7 ± 0.2	-2.6 ± 0.3	-3.1 ± 0.2	-2.8 ± 0.1	-2.7 ± 0.1	-2.4 ± 0.2	-2.7 ± 0.3
**Physical activity duration (min/day)**	242.7 ± 58.9	309.8 ± 57.6	353.1 ± 65.8	406.6 ± 86.1	315.8 ± 99.1	333.5 ± 90.5	333.4 ± 87.5	329.1 ± 84.0	327.9 ± 90.7
**Sex (%)**									
Men	7,801 (47.9%)	7,788 (47.9%)	7,769 (47.8%)	7,761 (47.8%)	7,801 (47.9%)	7,788 (47.9%)	7,769 (47.8%)	7,761 (47.8%)	31,119 (47.9%)
Women	8,495 (52.1%)	8,486 (52.1%)	8,469 (52.2%)	8,459 (52.2%)	8,495 (52.1%)	8,486 (52.1%)	8,469 (52.2%)	8,459 (52.2%)	33,909 (52.1%)
**Employment status (%)**									
Employed	11,662 (71.6%)	12,779 (78.5%)	12,965 (79.8%)	13,002 (80.2%)	11,814 (72.5%)	12,705 (78.1%)	12,926 (79.6%)	12,963 (79.9%)	50,408 (77.5%)
Not employed	4,506 (27.7%)	3,394 (20.9%)	3,175 (19.6%)	3,116 (19.2%)	4,346 (26.7%)	3,474 (21.3%)	3,217 (19.8%)	3,155 (19.5%)	14,192 (21.8%)
**Socioeconomic status** (ISEI-08) ^2^	49.9 ± 15.0	50.8 ± 15.2	50.3 ± 15.5	49.7 ± 15.8	46.8 ± 15.3	49.5 ± 15.3	51.2 ± 15.2	53.1 ± 15.1	50.2 ± 15.4
**Excess alcohol intake (%)** ^3^									
No	10,949 (67.2%)	10,422 (64.0%)	10,031 (61.8%)	9,923 (61.2%)	10,733 (65.9%)	10,381 (63.8%)	10,116 (62.3%)	10,095 (62.2%)	41,326 (63.6%)
Yes	4,883 (30.0%)	5,437 (33.4%)	5,808 (35.8%)	5,898 (36.4%)	5,027 (30.8%)	5,487 (33.7%)	5,730 (35.3%)	5,782 (35.6%)	22,025 (33.9%)
**Smoking status (%)**									
Never	6,988 (42.9%)	7,707 (47.4%)	7,768 (47.8%)	7,935 (48.9%)	6,737 (41.3%)	7,513 (46.2%)	7,824 (48.2%)	8,325 (51.3%)	30,399 (46.7%)
Former	5,026 (30.8%)	5,274 (32.4%)	5,344 (32.9%)	5,558 (34.3%)	5,336 (32.7%)	5,369 (33.0%)	5,288 (32.6%)	5,209 (32.1%)	21,202 (32.6%)
Current	3,854 (23.7%)	2,910 (17.9%)	2,747 (16.9%)	2,354 (14.5%)	3,737 (22.9%)	3,009 (18.5%)	2,753 (17.0%)	2,365 (14.6%)	11,864 (18.2%)
**Body mass index (kg/m** ^**2**^ **)**	28.0 ± 5.8	26.6 ± 4.7	25.9 ± 4.3	25.1 ± 3.9	28.0 ± 5.7	26.7 ± 4.8	25.9 ± 4.3	25.1 ± 3.9	26.4 ± 4.8
**Grip strength (kg)**	38.6 ± 11.9	39.1 ± 11.5	39.1 ± 11.5	39.2 ± 11.4	38.8 ± 11.9	39.2 ± 11.7	39.2 ± 11.5	38.8 ± 11.2	39.0 ± 11.6
**Sleep duration (hours/day)**	8.0 ± 1.2	7.8 ± 1.1	7.8 ± 1.1	7.7 ± 1.1	7.9 ± 1.2	7.8 ± 1.1	7.8 ± 1.1	7.8 ± 1.1	7.8 ± 1.1
**Sedentary time (hours/day)**	7.8 ± 4.0	7.0 ± 3.7	6.5 ± 3.6	5.9 ± 3.4	6.6 ± 3.9	6.6 ± 3.7	6.8 ± 3.8	7.1 ± 3.6	6.8 ± 3.8
**Cardiovascular disease (%)** ^4^									
No	9,791 (60.1%)	10,712 (65.8%)	11,152 (68.7%)	11,730 (72.3%)	9,769 (59.9%)	10,744 (66.0%)	11,200 (69.0%)	11,672 (72.0%)	43,386 (66.7%)
Yes	6,501 (39.9%)	5,555 (34.1%)	5,083 (31.3%)	4,482 (27.6%)	6,523 (40.0%)	5,524 (33.9%)	5,032 (31.0%)	4,542 (28.0%)	21,620 (33.2%)
**Diabetes (%)** ^4^									
No	14,810 (91.1%)	15,400 (94.8%)	15,486 (95.5%)	15,705 (96.9%)	14,834 (91.2%)	15,400 (94.8%)	15,523 (95.7%)	15,644 (96.6%)	61,402 (94.6%)
Yes	1,456 (8.9%)	846 (5.2%)	724 (4.5%)	499 (3.1%)	1,426 (8.8%)	849 (5.2%)	692 (4.3%)	558 (3.4%)	3,524 (5.4%)

Data are presented as age-standardised means ± standard deviations for continuous variables and number (percentage) for categorical variables.*mg*: milli-gravity unit.^1^ ENMO: Euclidean Norm Minus One acceleration metric derived from raw accelerometer data.^2^ ISEI-08: International Socioeconomic Index of Occupational Status: unitless index score of occupational socioeconomic status; range 10–89, with higher values indicating higher occupational status.^3^ AUDIT-C: Alcohol Use Disorders Identification Test-Consumption; thresholds ≥ 4 for men; ≥3 for women.^4^ Chronic diseases were self-reported physician diagnoses.

### Correlations and distribution of accelerometer-derived physical activity metrics

Correlations among physical activity metrics revealed several interrelationships (Additional file 1: Supplementary Figs. 3 and 4). Physical activity volume was moderately correlated with the intensity gradient (*r* = 0.55) and moderately to strongly correlated with the acceleration of the most active one to 120 min of the day (M1–M120; *r* = 0.67–0.92), which were themselves highly intercorrelated (*r* = 0.88–0.96; Additional file 1: Supplementary Fig. 4). The intensity gradient was likewise moderately to strongly correlated with the M1–M60 metrics (*r* = 0.61–0.85), and modestly correlated with M120 (*r* = 0.37). Correlations between the intensity gradient and acceleration during the most active continuous one to 120 min (M1-continuous–M120-continuous) declined with increasing bout duration (from *r* = 0.82 for M1-continuous to *r* = 0.11 for M120-continuous). Correlations between the intensity gradient and M-ratio, which captures whether activity was accumulated continuously or sporadically, were weaker overall (generally *r* < 0.5).

Across increasing accumulation of the most active daily minutes (M1–M120), acceleration decreased from 203.1 m*g* at M1 to 34.7 m*g* at M120. For the most active uninterrupted bouts of the same duration range (M1-continuous–M120-continuous), acceleration declined more steeply, from 149.0 m*g* to 5.7 m*g*, reflecting the reduced ability to sustain high acceleration over longer activity bouts. Correspondingly, activity patterns became increasingly fragmented as the activity window lengthened, reflected in declining M-ratio values from 0.7 to 0.2 (Additional file 1: Supplementary Table 3).

### Physical activity amount in relation to all-cause mortality

During a median follow-up of 8.0 years, corresponding to 514,297.5 person-years, 1,634 participants died (incidence rate = 3.2 per 1,000 person-years). Associations with mortality risk differed across indicators of physical activity.

**Fig. 1:**
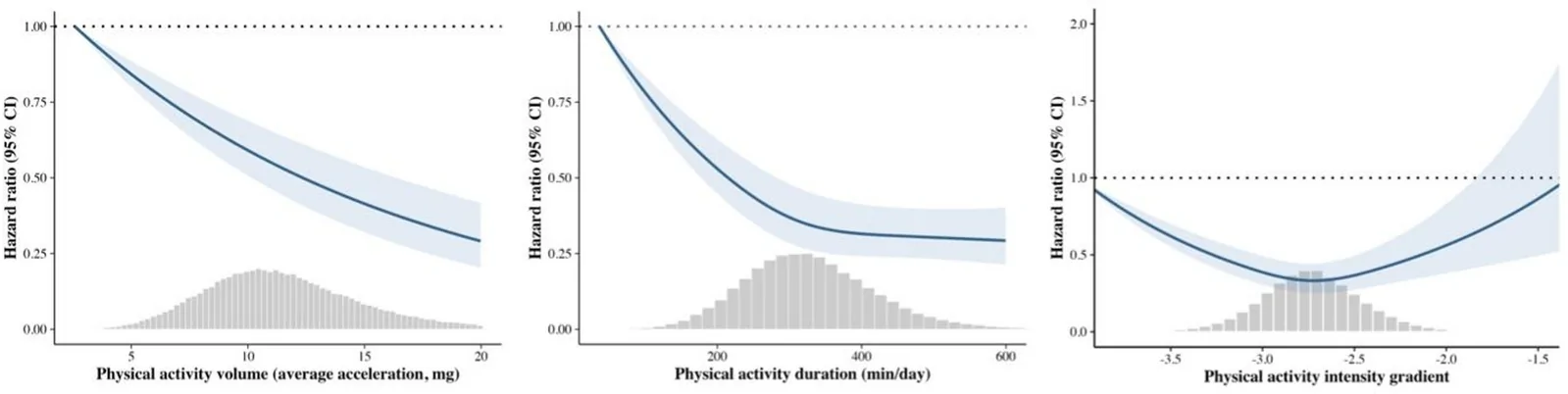
Physical activity volume, duration, and intensity gradient in relation to all-cause mortality among participants of the German NAKO. Solid lines represent hazard ratios and shaded areas 95% confidence intervals. All estimates were derived within the observed ranges of activity; no extrapolation beyond the empirical exposure distributions was performed. The histograms show the distribution of participants. All models were adjusted for a comprehensive set of covariates: sex, age, study region, socioeconomic status, employment status, alcohol consumption, smoking status, sedentary behavior, self-reported physician diagnosis of cardiovascular disease and diabetes, body mass index, handgrip strength, and sleep duration

In multivariable models, total physical activity volume displayed a linear inverse association with mortality (p_non−linearity_=0.127; Fig. 1). Relative to the 25th percentile (9.2 m*g*), activity levels at the 50th (11.2 m*g*) and 75th (13.7 m*g*) percentiles were associated with HRs of 0.87 (95% CI = 0.83–0.90) and 0.73 (95% CI = 0.67–0.80), respectively. Each 1 m*g* increase in average acceleration was associated with a 7% lower mortality risk (HR = 0.93; 95% CI = 0.91–0.95).

Total physical activity duration showed a non-linear inverse association with mortality (p_non−linearity_ <0.001; Fig. 1), with steeper risk reductions at lower and moderate durations and attenuation at higher durations. Compared with the 25th percentile (265.4 min/day), the 50th percentile (321.6 min/day) was associated with a 15% lower mortality risk (HR = 0.85; 95% CI = 0.81–0.88), with a more modest additional reduction at the 75th percentile (383.3 min/day; HR = 0.77; 95% CI = 0.72–0.82), reaching a plateau beyond that point (Fig. 1).

### Physical activity intensity in relation to all-cause mortality

The physical activity intensity gradient exhibited a shallow U-shaped association with mortality (p_non−linearity_<0.001; Fig. 1). Compared with the 25th percentile (-2.91), the 50th percentile (-2.74) was associated with a 7% lower mortality risk (HR = 0.93; 95% CI = 0.90–0.96), whereas the 75th percentile (-2.56) showed no association relative to the 25th percentile (HR = 0.98; 95% CI = 0.91–1.06).

Beyond the intensity gradient, the intensity achieved during the most active minutes of the day, irrespective of whether those minutes occurred consecutively or sporadically (M1–M120), were non-linearly inversely associated with mortality (Table 2 and Additional file 1: Supplementary Table 4), with associations weakening as duration increased. For illustration, comparing the 75th with the 25th percentile yielded HRs of 0.58 (95% CI = 0.53–0.63) for the most active minute of the day (M1) and 0.69 (95% CI = 0.65–0.74) for the most active 120 min of the day (M120).

**Table 2:** Intensity of the most active minutes (M1, M5, M15, M30, M60 and M120) and the most active continuous minutes (M1-continuous, M5-continuous, M15-continuous, M30-continuous, M60-continuous, M120-continuous) in relation to all-cause mortality among participants of the German NAKO

Metric	Q25	Q50	Q75	HR 50 vs. 25 (95% CI)	HR 75 vs. 25 (95% CI)	*P*_non−linear_
M1	171.3	203.1	247.2	0.73 (0.69–0.76)	0.58 (0.53–0.63)	< 0.0001
M5	138.8	164.8	197.4	0.76 (0.73–0.79)	0.63 (0.58–0.68)	< 0.0001
M15	107.8	131.0	159.4	0.78 (0.75–0.81)	0.66 (0.61–0.71)	< 0.0001
M30	81.7	101.7	126.4	0.79 (0.75–0.82)	0.67 (0.63–0.72)	< 0.0001
M60	52.2	66.4	83.7	0.78 (0.75–0.82)	0.69 (0.64–0.74)	< 0.0001
M120	26.8	34.7	44.3	0.79 (0.76–0.82)	0.69 (0.65–0.74)	< 0.0001
M1-continuous	117.2	149.0	187.2	0.81 (0.78–0.84)	0.71 (0.66–0.77)	< 0.0001
M5-continuous	60.7	96.3	139.3	0.85 (0.81–0.90)	0.78 (0.71–0.84)	0.0035
M15-continuous	24.0	44.4	79.6	0.86 (0.81–0.91)	0.75 (0.67–0.82)	0.0015
M30-continuous	12.0	22.6	45.4	0.85 (0.81–0.89)	0.68 (0.61–0.76)	< 0.0001
M60-continuous	6.8	10.7	20.5	0.86 (0.83–0.90)	0.68 (0.61–0.75)	< 0.0001
M120-continuous	3.9	5.7	8.5	0.92 (0.87–0.96)	0.83 (0.76–0.91)	0.0281

Comparison of the 50th and 75th percentile vs. the 25th percentile. Q25, Q50 and Q75 indicate the 25th, 50th, and 75th percentile of each metric. Models were adjusted for sex, age, study region, socioeconomic status, employment status, alcohol consumption, smoking status, sedentary behavior, self-reported physician diagnosis of cardiovascular disease and diabetes, body mass index, handgrip strength, and sleep duration

Results for M2-4, M10 and M20 (and respective continuous metrics) are provided in Additional file 1: Supplementary Table 4

### Temporal structure of physical activity in relation to all-cause mortality

Across all continuous bout durations from M1-continuous to M120-continuous, higher activity intensities were non-linearly inversely associated with mortality risks (all p_non−linearity_≤0.05; Table 2 and Additional file 1: Supplementary Table 4). When comparing the 50th with the 25th percentile, HRs ranged from 0.81 (95% CI = 0.78–0.84) for M1-continuous to 0.92 (95% CI = 0.87–0.96) for M120-continuous. The most pronounced inverse associations at the 75th percentile were observed for shorter and mid-duration bouts, including M1-continuous (HR = 0.71; 95% CI = 0.66–0.77), M30-continuous (HR = 0.68; 95% CI = 0.61–0.76), and M60-continuous (HR = 0.68, 95% CI = 0.61–0.75), whereas a slightly weaker association was observed for longer-term duration bouts of M120-continuous (HR = 0.83; 95% CI = 0.76–0.91).

**Fig. 2:**
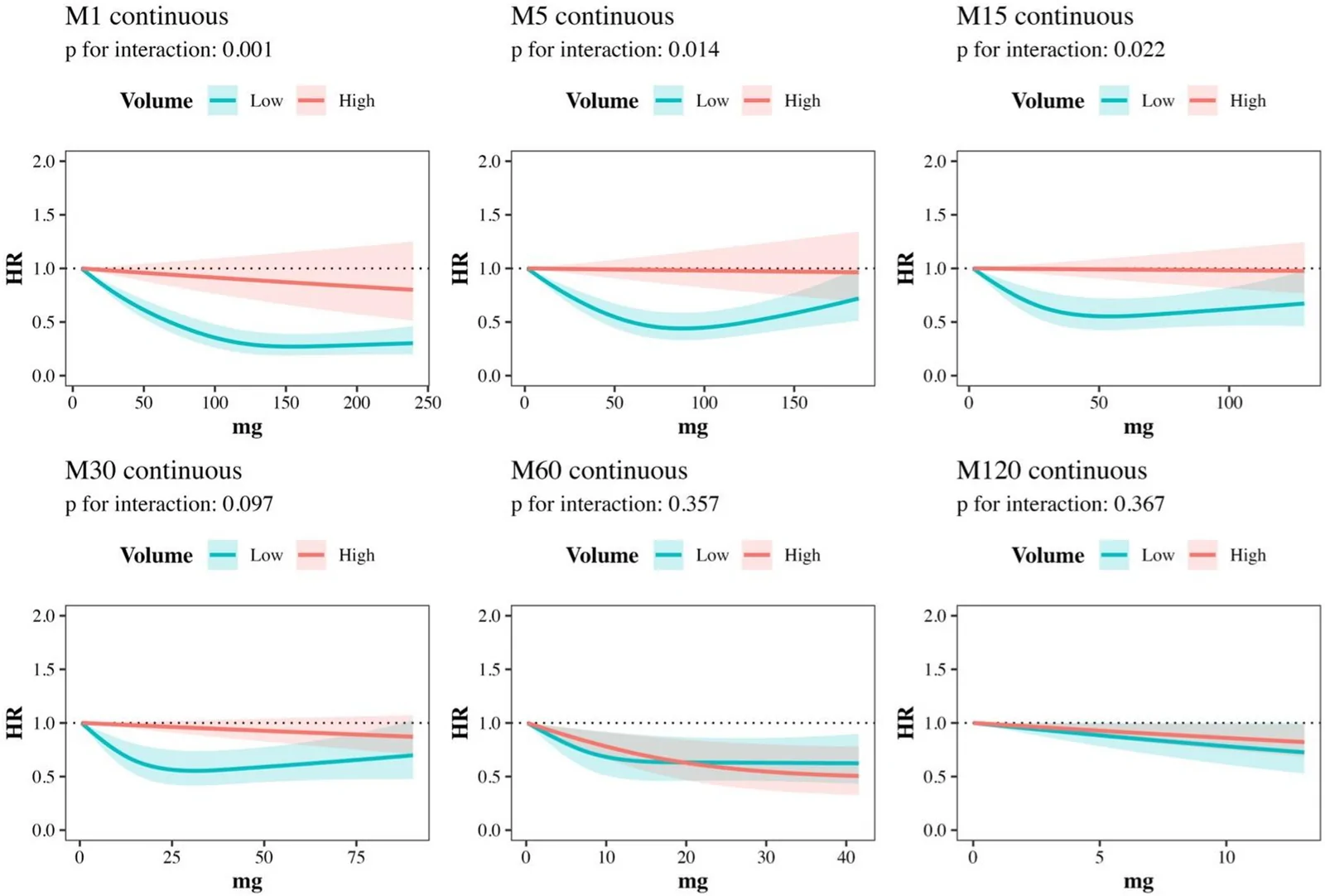
Intensity of the most active continuous minutes in relation to all-cause mortality by physical activity volume among participants of the German NAKO. Solid lines represent hazard ratios and shaded areas 95% confidence intervals. All models were adjusted for a comprehensive set of covariates: sex, age, study region, socioeconomic status, employment status, alcohol consumption, smoking status, sedentary behavior, self-reported physician diagnosis of cardiovascular disease and diabetes, body mass index, handgrip strength, and sleep duration. The volume cut-point was set at the median of physical activity volume (11.2 mg). For visualization purposes, the x-axis was truncated at the 90th percentile of the distribution for each bout duration. Results for M2-M4, M10 and M20 are provided in Additional file 1: Supplementary Figure 5

Associations between the intensity of the most active continuous minutes and mortality were modified by overall physical activity volume at shorter bout durations, with inverse associations being more pronounced among individuals with low overall activity volume (Fig. 2, Additional file 1: Supplementary Fig. 5). Differences in intensity-mortality relations between activity volume groups attenuated with increasing bout duration and were no longer apparent for 30, 60, and 120 continuous minutes (p_interaction_=0.097, 0.357, and 0.367, respectively).

**Fig. 3:**
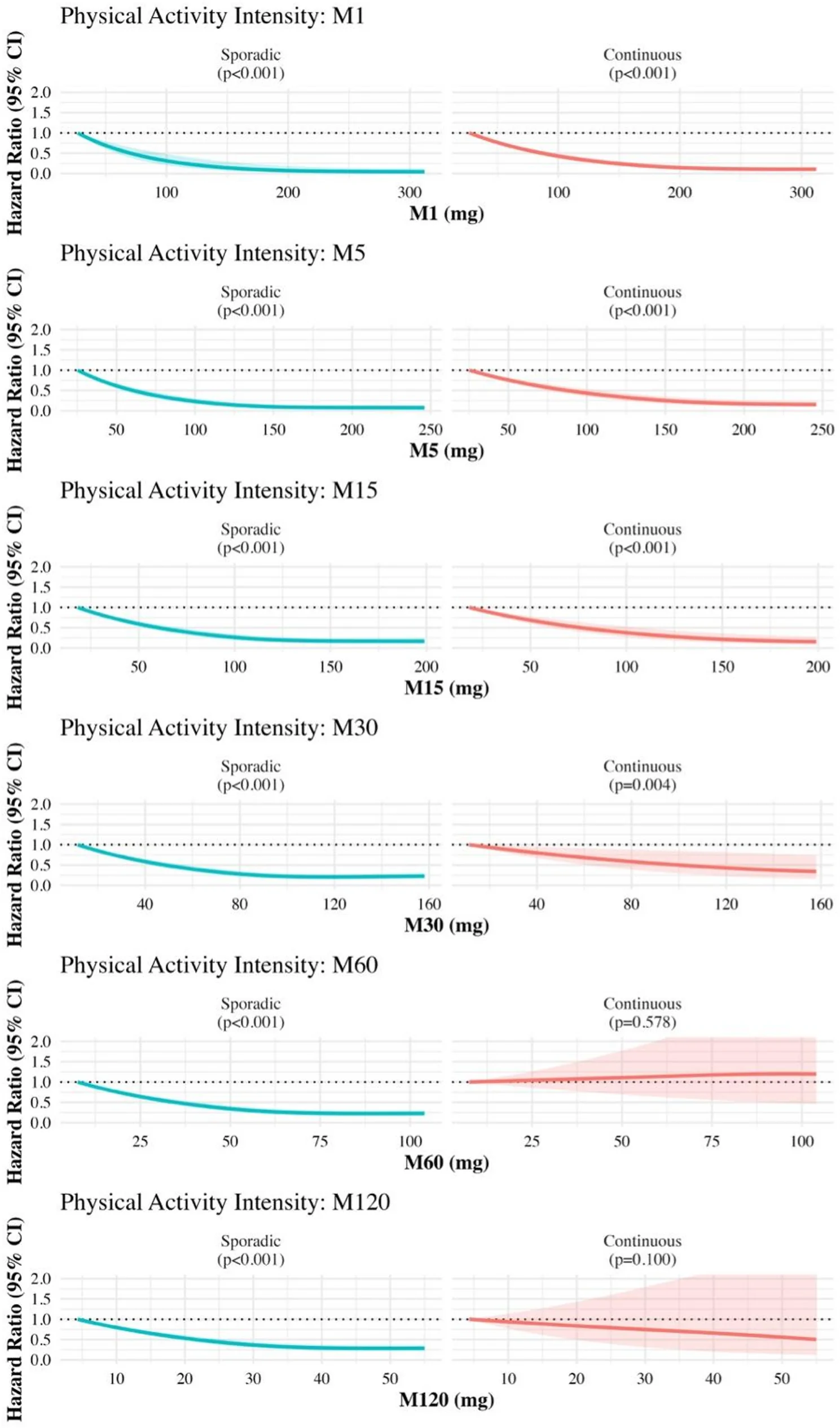
Intensity of the most active minutes in relation to all-cause mortality by accumulation pattern based on Mx-ratio (sporadic: Mx-ratio < 0.5; continuous: Mx-ratio ≥ 0.5) among participants of the German NAKO. Solid lines represent hazard ratios and shaded areas 95% confidence intervals. Analyses were stratified by accumulation pattern based on Mx-ratio metrics, categorized as sporadic Mx-ratio<0.5 or continuous: Mx-ratio≥0.5. All models were adjusted for a comprehensive set of covariates: sex, age, study region, socioeconomic status, employment status, alcohol consumption, smoking status, sedentary behavior, self-reported physician diagnosis of cardiovascular disease and diabetes, body mass index, handgrip strength, and sleep duration. Non-linearity was assessed using restricted cubic splines, with corresponding p-values indicated for each plot. To improve visualization, the x-axis displays values up to the 90th percentile of the respective distribution. Results for M2-M4, M10 and M20 are provided in Additional file 1: Supplementary Figure 6

Whereas the preceding analyses focused specifically on fully continuous bouts, we next examined whether the association for the broader Mx-metrics differed according to the extent to which these most active minutes were accumulated continuously vs. intermittently. Specifically, we conducted analyses of Mx-metrics stratified by the Mx-ratio, a measure that reflects the extent to which the most active minutes were accumulated continuously rather than intermittently (Fig. 3, Additional file 1: Supplementary Fig. 6). For lower Mx-ratio values (Mx-ratio < 0.5), corresponding to more intermittent activity accumulation, associations showed pronounced inverse relations across M1 to M120. In contrast, for higher Mx-ratio values (Mx-ratio ≥ 0.5), corresponding to more continuous activity accumulation, associations lost statistical significance at approximately 60 min.

### Interaction and sensitivity analyses

No statistically significant interactions were observed between physical activity metrics and covariates in relation to all-cause mortality after adjustment for multiple testing (Additional file 1: Supplementary Table 5), nor between activity volume and intensity gradient (p_interaction_=0.105). The proportional hazards assumption was met for the physical activity exposure in all models (Additional file 1: Supplementary Table 6). VIFs across physical activity metrics did not indicate problematic multicollinearity. For M30-, M60-, and M120-continuous, elevated VIFs (14.0–29.8) were observed among the spline basis terms of the exposure itself (Additional file 1: Supplementary Table 7).

Results were unchanged after excluding the first two years of follow-up (123 cases excluded; Additional file 1: Supplementary Fig. 7), excluding participants with prevalent cancer at baseline (4,162 cases excluded; Additional file 1: Supplementary Fig. 8), or removing cardiovascular disease, diabetes, and body mass index from the adjustment set (Additional file 1: Supplementary Fig. 9).

## Discussion

In this prospective cohort study of accelerometer-derived physical activity in more than 65,000 adults from NAKO, higher levels of physical activity were associated with lower all-cause mortality risk across multiple indicators of daily physical activity. While overall activity volume showed the most consistent association with mortality, activity intensity provided complementary information beyond volume, and its association with mortality depended on how activity was accumulated, with stronger inverse associations for intermittently accumulated activity and for shorter (≤ 60 min) compared with longer (120 min) continuous durations.

### Physical activity amount and all-cause mortality

Total daily physical activity volume showed a linear inverse association with mortality, with each 1 m*g* increase associated with a 7% lower mortality risk. A difference of 1 m*g* in acceleration roughly corresponds to several minutes of additional brisk walking per day [27], although the exact translation depends on movement patterns and device calibration. Previous studies have also reported inverse relationships across tertiles of activity volume [3], when activity volume was expressed as average acceleration [5] or physical activity energy expenditure [28], and when average acceleration was examined in relation to life expectancy [29]. These findings suggest that the overall volume of daily movement behaviour plays a central role in the association between physical activity and mortality.

Total daily physical activity duration showed a non-linear inverse association with mortality, with risk declining steeply at lower activity durations and attenuating at higher durations. Previous accelerometer-based studies have also reported inverse associations between activity duration and mortality but have typically examined time spent in specific activity intensities, such as light, moderate, or vigorous physical activity [30, 31], or have been conducted in clinical populations [32]. In contrast, our analysis quantified total activity duration across intensities among participants of a population-based large-scale study, an approach that may reduce dependence on intensity cut-points and aligns with emerging public health paradigms emphasizing overall physical activity [33]. Taken together, accumulating more daily movement time, irrespective of intensity, may confer substantial health benefits, with progressively smaller gains observed beyond moderate daily durations (beyond approximately 320 min per day).

### Physical activity intensity and all-cause mortality

The physical activity intensity gradient, summarizing the distribution of activity intensities across the day, showed a shallow U-shaped association with mortality risk, with the lowest risk observed for activity profiles characterized by moderate overall activity together with some exposure to higher-intensity activity. Estimates at the upper end of the intensity gradient distribution should be interpreted cautiously, as relatively few participants exhibited such activity profiles and estimates in this range were therefore less stable. Previous studies have reported largely linear inverse associations between the intensity gradient and mortality, whereby greater relative contributions of higher-intensity activity were associated with progressively lower risk [5, 9].

Beyond the intensity gradient, the most active minutes of the day, irrespective of whether these minutes were accumulated sporadically or continuously (Mx-metrics), were inversely associated with mortality across durations from one to 120 min, with associations becoming progressively attenuated as duration increased. Previous studies have also reported inverse associations with mortality but have largely focused on very short bursts of higher-intensity activity lasting up to approximately two minutes [7] or a limited set of metrics extending to about 20 min [9]. By investigating durations of up to 120 min, our analysis provides a more comprehensive assessment of associations between physical activity intensity and mortality as higher activity intensities are sustained for longer periods of daily physical activity.

### Physical activity accumulation patterns and all-cause mortality

Our analyses of Mx-continuous-metrics demonstrated that the inverse association between sustained activity intensity and mortality was modified by total activity volume. Among individuals with lower overall activity volume, higher intensity of continuous bouts was associated with lower mortality across bout durations, although associations weakened with increasing bout duration. Among those with higher overall activity volume, no clear associations were observed at shorter bout durations, whereas inverse associations emerged at longer durations, resulting in more similar intensity–mortality associations across activity volume groups. These results extend and refine previous research that reported no association between bout duration and mortality after adjusting for volume [34, 35]. In contrast to such adjustment-based approaches, our stratified analyses suggest that associations with activity intensity within continuous bouts differ by overall activity volume, particularly for shorter bouts. Subsequent work has shown that, compared with bouts shorter than one minute, bouts lasting at least one minute were associated with lower mortality risk, whereas bouts longer than about five to ten minutes provided little additional benefit [36].

Stratification by the Mx-ratio further refined these results by showing that the association of higher-intensity activity with lower mortality differed according to whether the most active minutes were accumulated more intermittently or more continuously. When the most active minutes were accumulated predominantly intermittently, inverse associations were evident across durations from M1 to M120, whereas for predominantly continuous accumulation, associations were attenuated and no longer evident at approximately 60 min. This attenuation at longer durations for predominantly continuous accumulation may reflect limits in the ability to sustain higher intensities over prolonged uninterrupted periods, as well as reduced variability and precision in these exposure metrics at the upper end of the duration distribution. Consistent with prior work suggesting that the optimal accumulation pattern may depend on activity intensity, with more sustained accumulation potentially favouring moderate intensities and more intermittent accumulation favouring higher intensities [9], our findings indicate that intermittent accumulation may better preserve the association of higher-intensity activity with lower mortality across longer daily durations.

Taken together, our findings from the volume-stratified Mx-continuous analyses and the Mx-ratio stratification indicate that the associations of activity intensity and mortality are shaped jointly by overall activity volume, duration, and accumulation pattern. Higher intensity achieved during continuous bouts was more strongly associated with lower mortality risk among individuals with lower versus higher overall activity volume at shorter bout durations, whereas intensity–mortality associations were more similar across activity volume groups at longer durations. At the same time, across the broader Mx-metrics, more intermittent accumulation was associated with inverse associations across a wider duration range than more continuous accumulation. These complementary findings suggest that how higher-intensity activity is distributed across the day may be as relevant as how much of it is performed. Our observational findings indicate that, if confirmed in future experimental studies, intermittent accumulation may emerge as a particularly relevant pattern for sustaining beneficial intensity exposures over longer periods of daily activity.

The observed inverse associations of higher activity volume and intensity may be mediated through improvements in cardiometabolic, immune, and brain health [37], as well as through processes related to healthy ageing [38]. Our findings are consistent with the notion that substantial physiological benefits of physical activity may be achieved through moderate intensities when overall activity volume is sufficient, without requiring extreme exertion. Future studies investigating cause-specific mortality may provide further insight into potential mechanisms underlying the observed associations.

From a public health perspective, our results support strategies that encourage greater daily movement behaviour across a wide range of indicators of physical activity (intensity and accumulation pattern), as apparent benefits were not restricted to a specific structure of activity bouts. These observations support current physical activity guidelines emphasizing that ‘every move counts’ or ‘sit less, move more’ and that benefits accrue across a wide range of activity patterns and intensities [33, 39]. Cause-specific mortality will be an important endpoint for future studies to assess the role of volume, intensity, and accumulation patterns to inform targeted public health guidance.

### Strengths and limitations

Several strengths of our study merit consideration. The large sample size provided substantial statistical power to explore multiple indicators of physical activity in relation to risk of all-cause mortality. The use of high-resolution, cut-point-free accelerometer-based data enabled objective measurement of movement behaviour across the full physical activity intensity spectrum, including short and sporadic activity episodes. In addition, the analysis incorporated complementary indicators that characterize distinct aspects of daily physical activity, including volume, duration, intensity distribution, peak activity intensities, and accumulation patterns. This multidimensional approach enabled a comprehensive examination of how different characteristics of daily physical activity relate to all-cause mortality risk. Extensive sensitivity analyses, including the exclusion of the first two years of follow-up and participants with prevalent disease, yielded similar results, suggesting that the observed associations are unlikely to be explained by reverse causation or undiagnosed illness.

Nevertheless, our study has certain limitations. Physical activity was assessed during a single baseline period and may not capture changes over time due to ageing, retirement, incident disease, or other health- and lifestyle-related factors. Accordingly, our findings primarily reflect associations with physical activity patterns at baseline, while subsequent changes in physical activity could not be accounted for. Hip-worn accelerometers may underestimate certain activities, such as cycling, swimming, or upper-body movement, potentially leading to exposure misclassification. Device-based measures also do not distinguish between physical activity domains (e.g., occupational, household, transportation, or leisure-time), and higher activity volume or intensity may partly reflect occupational activity, which has been associated with adverse health outcomes [40]. Although we adjusted for employment status and socioeconomic status, this cannot fully substitute for direct information on activity domains, and residual confounding remains possible despite adjustment for a wide range of covariates identified through directed acyclic graphs. Confounding by underlying health status or physical fitness may also persist, although adjustment for grip strength, body mass index, self-reported physician diagnosis of chronic diseases, and multiple lifestyle factors likely reduced this possibility. Although the baseline response proportion was modest, selection bias is unlikely to have materially affected exposure and endpoint associations, as has been demonstrated in other large prospective cohort studies with similar participation rates [41]. Lastly, the observational nature of our study precludes causal interpretation.

## Conclusions

In conclusion, higher daily physical activity across multiple indicators was associated with lower risk of all-cause mortality. Overall activity volume showed the most consistent association, underscoring the importance of greater total daily movement. At the same time, intensity and accumulation patterns provided complementary information. Higher-intensity activity was more strongly associated with lower risk of all-cause mortality when accumulated intermittently and over short durations than when sustained over longer continuous periods. At shorter bout durations, associations between activity intensity and mortality were stronger among individuals with lower overall activity volume compared with those with higher overall activity volume, whereas differences in these associations diminished at longer durations. Given the observational design and potential residual confounding by underlying health and fitness, these associations should not be interpreted causally. These findings support a multidimensional perspective on physical activity that considers total movement behaviour alongside intensity and accumulation pattern.

## Supplementary Information

Below is the link to the electronic supplementary material.

Supplementary Material 1

Supplementary Material 2

## Data Availability

Researchers based in the European Union (EU) may request access to the NAKO dataset via the official portal at http://transfer.nako.de/. Supplementary materials related to this study are available from the corresponding author upon request.

## Abbreviations

AUDIT-C: Alcohol Use Disorder Identification Test-Consumption
CI: Confidence interval
HR: Hazard ratio
MET: Metabolic equivalent of task
NAKO: German National Cohort (NAKO Gesundheitsstudie)
VIF: Variance inflation factor
