# Supplementary material for: Daily physical activity volume, intensity, and accumulation patterns in relation to all-cause mortality in the German National Cohort (NAKO)
Source: BMC Med. 2026 Nov 10;24:636. doi: 10.1186/s12916-026-05385-8 (PMC13756916; doi:10.1186/s12916-026-05385-8)
Supplement: Supplementary file 1 [file 12916_2026_5385_MOESM1_ESM.docx]

**Daily physical activity volume, intensity, and accumulation patterns in relation to all-cause mortality in the German National Cohort (NAKO)**

**Supplementary material**

[Supplementary Figure 1.	Flowchart of participant inclusion](#_Toc238627910)

[Supplementary Figure 2.	Directed acyclic graph for confounder selection](#_Toc238627911)

[Supplementary Figure 3.	Correlation matrix for all physical activity exposures](#_Toc238627912)

[Supplementary Figure 4.	Correlation matrix for selected physical activity exposures](#_Toc238627913)

[Supplementary Figure 5.	Intensity of the most active continuous minutes in relation to all-cause mortality by physical activity volume.](#_Toc238627914)

[Supplementary Figure 6.	Accumulation patterns of physical activity intensity in relation to mortality](#_Toc238627915)

[Supplementary Figure 7.	Sensitivity Analysis 1: Associations between physical activity volume and intensity with mortality after exclusion of the first two years of follow-up.](#_Toc238627916)

[Supplementary Figure 8.	Sensitivity Analysis 2: Association between physical activity duration, volume and intensity with mortality after excluding prevalent cancer cases.](#_Toc238627917)

[Supplementary Figure 9. Sensitivity Analysis 3: Associations between physical activity volume and intensity with mortality after removal of cardiovascular diseases, diabetes, and body mass index from the covariate adjustment set](#_Toc238627918)

[Supplementary Table 1.	Physical activity metrics.](#_Toc238627919)

[Supplementary Table 2.	Covariate information.](#_Toc238627920)

[Supplementary Table 3.	Distribution of M-metrics across durations of the most active minutes among participants of the German NAKO](#_Toc238627921)

[Supplementary Table 4.	Intensity of the most active minutes (M1–M120) and the most active continuous minutes (M1-continuous–M120-continuous) in relation to all-cause mortality among participants of the German NAKO.](#_Toc238627922)

[Supplementary Table 5.	Interaction tests between physical activity volume and intensity with all covariates](#_Toc238627923)

[Supplementary Table 6.	Proportional hazards assumption diagnostics (Schoenfeld residuals) for Cox models of core physical activity metrics.](#_Toc238627924)

[Supplementary Table 7.	Variance inflation factors (VIFs) for Cox proportional hazards models of core physical activity metrics.](#_Toc238627925)

**Corresponding authors:**

Patricia Fromherz, Mail: patricia.fromherz@ur.de, Department of Epidemiology and Preventive Medicine, University of Regensburg, Regensburg, Germany

Michael J. Stein, Mail: michael.stein@helmholtz-munich.de, Institute of Epidemiology, Helmholtz Zentrum München, German Research Center for Environmental Health (GmbH), Neuherberg, Germany

# Supplementary Figure 1. Flowchart of participant inclusion

# Supplementary Figure 2. Directed acyclic graph for confounder selection

# Supplementary Figure 3. Correlation matrix for all physical activity exposures

TPA: Total physical activity duration; AvgAcc: Average acceleration; IG: Intensity gradient

# Supplementary Figure 4. Correlation matrix for selected physical activity exposures

# Supplementary Figure 5. Intensity of the most active continuous minutes in relation to all-cause mortality by physical activity volume.

Solid lines represent hazard ratios and shaded areas 95% confidence intervals. All models were adjusted for a comprehensive set of covariates: sex, age, study region, socioeconomic status, employment status, alcohol consumption, smoking status, sedentary behavior, self-reported physician diagnosis of cardiovascular disease and diabetes, body mass index, handgrip strength, and sleep duration. The volume cut-point was set at the median of physical activity volume (11.2 m*g*). For visualization purposes, the x-axis was truncated at the 90th percentile of the distribution for each bout duration.

# Supplementary Figure 6. Accumulation patterns of physical activity intensity in relation to mortality

Note: MX metrics represent the intensity of a person’s most active X minutes, irrespective of the accumulation pattern. M120 “Continuous” results are not shown because only 46 deaths occurred within the corresponding exposure range, resulting in unstable estimates.

# Supplementary Figure 7. Sensitivity Analysis 1: Associations between physical activity volume and intensity with mortality after exclusion of the first two years of follow-up.

# Supplementary Figure 8. Sensitivity Analysis 2: Association between physical activity duration, volume and intensity with mortality after excluding prevalent cancer cases.

# Supplementary Figure 9. Sensitivity Analysis 3: Associations between physical activity volume and intensity with mortality after removal of cardiovascular diseases, diabetes, and body mass index from the covariate adjustment set

## Supplementary Table 1. Physical activity metrics.

| **Metric** | **Short description** | **Calculation** |
| --- | --- | --- |
| Volume | Mean acceleration magnitude across a full day | Average ENMO, first calculated per day and then averaged across all valid days. |
| Duration | Daily physical activity minutes | Average total physical activity per day, calculated as the sum of mean daily light, moderate and vigorous intensity physical activity. |
| Intensity gradient | Intensity distribution across a full day. Higher values indicate more time spent at higher intensity levels. | Calculated as the slope of the linear regression fitted to the log-transformed time and acceleration axes. |
| MX | Intensity of a person’s most active X minutes, irrespective of the accumulation pattern. | Calculated as the acceleration above which a person’s most active X minutes are accumulated, irrespective of the accumulation pattern. |
| MX_continuous_ | Intensity of a person’s most active X minutes accumulated in a continuous bout. | Calculated as the 25^th^ percentile of the acceleration during the most active continuous X minutes of the day. |
| MX_ratio_ | Ratio of MX_continuous_ and MX, indicating fragmentation of the most active minutes per day. | Calculated as the ratio of the intensity of the most active continuous X min (MX_continuous_) to the intensity of the same duration of most active minutes accumulated (MX) across a 24-h day |
| Participants were instructed to wear an ActiGraph accelerometer (GT3X+, wGT3X+, and wGT3X-BT; ActiGraph, Pensacola, FL, USA) on the right hip continuously for seven consecutive days starting the day after their baseline study visit, removing it only for water-based activities exceeding 30 minutes. Raw triaxial acceleration was recorded with a sampling frequency of 100 Hz and Idle Sleep Mode disabled.  Raw acceleration data were processed using GGIR (version 3.2-9) in R (version 4.3.1) on a high-performance computing cluster, including auto-calibration against local gravity [15], non-wear detection, sleep detection [17, 21] and intensity classification [18, 20]. Processing with GGIR was performed using 5-second epochs and Euclidean Norm Minus One (ENMO). Previously defined ENMO thresholds were used to define sedentary (<8mg), light (8-69mg), moderate (69-259mg) and vigorous (>259mg) intensity physical activity [18, 20]. Primary sleep windows were identified using the Heuristic algorithm looking at Distribution of Change in Z-Angle (HDCZA), with optimized parameters for hip-worn accelerometers [17, 19, 21]. | | |

## Supplementary Table 2. Covariate information.

| **Covariate** | **Categorization** | **Assessment** |
| --- | --- | --- |
| Sex | Women, men | Touchscreen-questionnaire |
| Age group | 5-year categories | Touchscreen-questionnaire |
| Study center region | South-east, south-west, north-east, north-west, west, central, Berlin | Touchscreen-questionnaire |
| Socioeconomic status | continuous | Interview; International Socioeconomic Index of Occupational Status: occupational socioeconomic status index based on the relationship between education, occupation, and income – derived from International Social Survey Programme data; range: 10-89 |
| Employment status | Employed, unemployed | Interview |
| Alcohol consumption | Risky alcohol consumption: yes or no | Alcohol Use Disorder Identification Test-Consumption [AUDIT-C] score ≥4 for men, ≥3 for women |
| Smoking status | Never, former, current | Touchscreen-questionnaire |
| Cardiovascular disease | Yes, no | Self-reported physician diagnosis via interview |
| Diabetes | Yes, no | Self-reported physician diagnosis via interview |
| Body-Mass-Index | kg/m**^2^**, continuous | Measured via bioelectrical impedance analysis |
| Hand grip strength | kg, continuous | Measured; Maximum value recorded during handgrip strength testing from all measurements performed on either side |
| Device-based sedentary time | Hours per day, continuous | Average sedentary time per day, first calculated per day and then averaged across all valid days. |
| Device-based sleep duration | Hours per night, continuous | Average sleep duration per day, first calculated per day and then averaged across all valid days. |

## Supplementary Table 3. Distribution of M-metrics across durations of the most active minutes among participants of the German NAKO

| **Duration of the most active minutes (min)** | **M** | **M**-**continuous** | **M**-**ratio** |
| --- | --- | --- | --- |
| Definition | Acceleration threshold above which the most active minutes of the day are accumulated, irrespective of whether they occur continuously or sporadically | 25^th^ percentile of acceleration within the most active continuous minutes of the day, ensuring ≥75% of the bout occurs above this threshold | Ratio of the most active continuous minutes to the most active minutes overall (M-continuous/M), reflecting activity fragmentation |
| 1 | 203.1 (171.3, 247.2) m*g* | 149.0 (117.2, 187.2) m*g* | 0.7 (0.6, 0.8) |
| 2 | 186.8 (158.2, 224.6) m*g* | 131.2 (96.9, 170.5) m*g* | 0.7 (0.6, 0.8) |
| 3 | 177.5 (150.0, 212.5) m*g* | 117.6 (81.6, 158.7) m*g* | 0.7 (0.5, 0.8) |
| 4 | 170.5 (143.9, 204.0) m*g* | 106.4 (70.1, 148.5) m*g* | 0.6 (0.5, 0.8) |
| 5 | 164.8 (138.8, 197.4) m*g* | 96.3 (60.7, 139.3) m*g* | 0.6 (0.4, 0.8) |
| 10 | 144.9 (120.6, 174.8) m*g* | 62.7 (35.3, 103.5) m*g* | 0.4 (0.3, 0.6) |
| 15 | 131.0 (107.8, 159.4) m*g* | 44.4 (24.0, 79.6) m*g* | 0.4 (0.2, 0.6) |
| 20 | 119.7 (97.6, 146.9) m*g* | 33.9 (17.7, 64.2) m*g* | 0.3 (0.2, 0.5) |
| 30 | 101.7 (81.7, 126.4) m*g* | 22.6 (12.0, 45.4) m*g* | 0.2 (0.1, 0.4) |
| 60 | 66.4 (52.2, 83.7) m*g* | 10.7 (6.8, 20.5) m*g* | 0.2 (0.1, 0.3) |
| 120 | 34.7 (26.8, 44.3) m*g* | 5.7 (3.9, 8.5) m*g* | 0.2 (0.1, 0.2) |
| m*g*: milli-gravity unit.  Values are median (interquartile range). | | | |

## Supplementary Table 4. Intensity of the most active minutes (M1–M120) and the most active continuous minutes (M1-continuous–M120-continuous) in relation to all-cause mortality among participants of the German NAKO.

| **Metric** | **Q25** | **Q50** | **Q75** | **HR 50 vs 25 (95% CI)** | **HR 75 vs 25 (95% CI)** | **P_non-linear_** |
| --- | --- | --- | --- | --- | --- | --- |
| M2 | 158.2 | 186.8 | 224.6 | 0.74 (0.71-0.77) | 0.60 (0.56-0.65) | <.0001 |
| M3 | 150.0 | 177.5 | 212.5 | 0.75 (0.72-0.78) | 0.62 (0.57-0.66) | <.0001 |
| M4 | 143.9 | 170.5 | 204.0 | 0.75 (0.72-0.78) | 0.63 (0.58-0.67) | <.0001 |
| M10 | 120.6 | 144.9 | 174.8 | 0.77 (0.74-0.80) | 0.65 (0.60-0.70) | <.0001 |
| M20 | 97.6 | 119.7 | 146.9 | 0.78 (0.75-0.81) | 0.66 (0.62-0.71) | <.0001 |
| M2-continuous | 96.9 | 131.2 | 170.5 | 0.83 (0.79-0.87) | 0.75 (0.69-0.80) | <.0001 |
| M3-continuous | 81.6 | 117.6 | 158.7 | 0.84 (0.80-0.88) | 0.76 (0.71-0.83) | 0.0001 |
| M4-continuous | 70.1 | 106.4 | 148.5 | 0.85 (0.81-0.90) | 0.78 (0.72-0.84) | 0.0015 |
| M15-continuous | 24.0 | 44.4 | 79.6 | 0.86 (0.81-0.91) | 0.75 (0.67-0.82) | 0.0015 |
| M20-continuous | 17.7 | 33.9 | 64.2 | 0.86 (0.81-0.91) | 0.72 (0.65-0.81) | 0.0008 |

## Supplementary Table 5. Interaction tests between physical activity volume and intensity with all covariates

| **Variable** | **Physical activity duration** | **Physical activity volume** | **Physical activity intensity gradient** |
| --- | --- | --- | --- |
| Employment status | 0.8167 | 0.3324 | 0.7504 |
| Socio-economic status | 0.4157 | 0.6087 | 0.4157 |
| Alcohol consumption | 0.0703 | 0.3074 | 0.5464 |
| Smoking status | 0.8735 | 0.9194 | 0.8127 |
| Sleep duration | 0.6497 | 0.8735 | 0.4157 |
| Body mass index | 0.6087 | 0.8232 | 0.6497 |
| Handgrip strength | 0.7504 | 0.0703 | 0.8167 |
| Sedentary behaviour | 0.3584 | 0.3637 | 0.8167 |
| Cardiovascular diseases | 0.6087 | 0.1011 | 0.1011 |
| Diabetes | 0.9202 | 0.8949 | 0.8993 |
| Numbers are P for multiplicative interaction obtained from Wald tests after false discovery rate correction. No interactions remained statistically significant after false-discovery-rate correction. | | | |

## Supplementary Table 6. Proportional hazards assumption diagnostics (Schoenfeld residuals) for Cox models of core physical activity metrics.

| **Physical activity metric** | **Exposure term, p**  **(mean)** | **Exposure term, p**  **(across imputations)** | **Global test, p**  **(mean)** | **Global test, p**  **(across imputations)** | **Imputations with**  **global p<0.05** | **Employment status**  **term, p (mean)** | **Employment status term,**  **p (across imputations)** |
| --- | --- | --- | --- | --- | --- | --- | --- |
| Physical activity duration | 0.091 | 0.075-0.107 | 0.058 | 0.021-0.102 | 4/10 | 0.006 | 0.002-0.011 |
| Physical activity volume | 0.811 | 0.756-0.857 | 0.168 | 0.071-0.268 | 0/10 | 0.012 | 0.005-0.018 |
| Intensity gradient | 0.456 | 0.420-0.528 | 0.104 | 0.042-0.192 | 2/10 | 0.008 | 0.003-0.013 |
| M1 | 0.585 | 0.552-0.631 | 0.191 | 0.096-0.310 | 0/10 | 0.011 | 0.005-0.017 |
| M5 | 0.368 | 0.347-0.407 | 0.152 | 0.070-0.252 | 0/10 | 0.012 | 0.006-0.019 |
| M15 | 0.273 | 0.257-0.305 | 0.129 | 0.057-0.216 | 0/10 | 0.012 | 0.005-0.018 |
| M30 | 0.337 | 0.318-0.373 | 0.131 | 0.056-0.214 | 0/10 | 0.010 | 0.004-0.016 |
| M60 | 0.529 | 0.502-0.562 | 0.136 | 0.056-0.223 | 0/10 | 0.008 | 0.003-0.013 |
| M120 | 0.951 | 0.897-0.995 | 0.145 | 0.058-0.235 | 0/10 | 0.008 | 0.003-0.013 |
| M1-continuous | 0.280 | 0.255-0.315 | 0.101 | 0.043-0.175 | 1/10 | 0.006 | 0.003-0.010 |
| M5-continuous | 0.060 | 0.055-0.070 | 0.034 | 0.011-0.061 | 8/10 | 0.003 | 0.001-0.005 |
| M15-continuous | 0.139 | 0.127-0.154 | 0.046 | 0.016-0.087 | 7/10 | 0.002 | 0.001-0.004 |
| M30-continuous | 0.618 | 0.587-0.653 | 0.088 | 0.034-0.161 | 1/10 | 0.002 | 0.001-0.004 |
| M60-continuous | 0.363 | 0.338-0.373 | 0.070 | 0.026-0.130 | 2/10 | 0.002 | 0.001-0.004 |
| M120-continuous | 0.523 | 0.497-0.555 | 0.077 | 0.029-0.143 | 2/10 | 0.002 | 0.001-0.004 |
| *Schoenfeld residual tests (cox.zph) were performed separately for each of 10 imputed datasets; p-values are summarized as the mean and range across imputations. "Global test" refers to the omnibus test across all model terms combined. "Exposure term" refers to the test for the physical activity metric of primary interest in each model.*  *A p-value <0.05 indicates evidence against the proportional hazards assumption for the respective term. The proportional hazards assumption was met for the physical activity exposure of interest in all 15 models (all exposure-term p>0.05, pooled across imputations). Violations were observed for employment status in several models, contributing to violations in the global test.* | | | | | | | |

## Supplementary Table 7. Variance inflation factors (VIFs) for Cox proportional hazards models of core physical activity metrics.

| **Metric** | **Term** | **Mean VIF** | **Min VIF** | **Max VIF** |
| --- | --- | --- | --- | --- |
| Intensity gradient | Body mass index | 1.2 | 1.2 | 1.2 |
| Intensity gradient | CVD (yes) | 1.1 | 1.1 | 1.1 |
| Intensity gradient | Diabetes (no) | 1.1 | 1.1 | 1.1 |
| Intensity gradient | Employment (unemployed) | 1.1 | 1.1 | 1.1 |
| Intensity gradient | Grip strength | 1.1 | 1.1 | 1.1 |
| Intensity gradient | Intensity gradient | 2.4 | 2.4 | 2.4 |
| Intensity gradient | Intensity gradient' | 2.2 | 2.2 | 2.2 |
| Intensity gradient | Risky alcohol use (yes) | 1.0 | 1.0 | 1.0 |
| Intensity gradient | Sedentary behaviour | 1.1 | 1.1 | 1.1 |
| Intensity gradient | Sleep duration | 1.0 | 1.0 | 1.0 |
| Intensity gradient | Smoking status (current) | 1.5 | 1.5 | 1.5 |
| Intensity gradient | Smoking status (former) | 1.4 | 1.4 | 1.4 |
| Intensity gradient | Socioeconomic status | 1.1 | 1.1 | 1.1 |
| M1 | Body mass index | 1.2 | 1.2 | 1.2 |
| M1 | CVD (yes) | 1.1 | 1.1 | 1.1 |
| M1 | Diabetes (no) | 1.1 | 1.1 | 1.1 |
| M1 | Employment (unemployed) | 1.1 | 1.1 | 1.1 |
| M1 | Grip strength | 1.1 | 1.1 | 1.1 |
| M1 | M1 | 3.6 | 3.6 | 3.6 |
| M1 | M1' | 3.1 | 3.1 | 3.1 |
| M1 | Risky alcohol use (yes) | 1.0 | 1.0 | 1.0 |
| M1 | Sedentary behaviour | 1.1 | 1.1 | 1.1 |
| M1 | Sleep duration | 1.0 | 1.0 | 1.1 |
| M1 | Smoking status (current) | 1.5 | 1.5 | 1.5 |
| M1 | Smoking status (former) | 1.4 | 1.4 | 1.4 |
| M1 | Socioeconomic status | 1.1 | 1.1 | 1.1 |
| M1 continuous | Body mass index | 1.2 | 1.2 | 1.2 |
| M1 continuous | CVD (yes) | 1.1 | 1.1 | 1.1 |
| M1 continuous | Diabetes (no) | 1.1 | 1.1 | 1.1 |
| M1 continuous | Employment (unemployed) | 1.1 | 1.1 | 1.1 |
| M1 continuous | Grip strength | 1.1 | 1.0 | 1.1 |
| M1 continuous | M1 continuous | 3.6 | 3.6 | 3.6 |
| M1 continuous | M1 continuous' | 3.3 | 3.3 | 3.3 |
| M1 continuous | Risky alcohol use (yes) | 1.0 | 1.0 | 1.0 |
| M1 continuous | Sedentary behaviour | 1.1 | 1.1 | 1.1 |
| M1 continuous | Sleep duration | 1.0 | 1.0 | 1.0 |
| M1 continuous | Smoking status (current) | 1.5 | 1.5 | 1.5 |
| M1 continuous | Smoking status (former) | 1.4 | 1.4 | 1.4 |
| M1 continuous | Socioeconomic status | 1.1 | 1.1 | 1.1 |
| M120 | Body mass index | 1.2 | 1.2 | 1.2 |
| M120 | CVD (yes) | 1.1 | 1.1 | 1.1 |
| M120 | Diabetes (no) | 1.1 | 1.1 | 1.1 |
| M120 | Employment (unemployed) | 1.1 | 1.1 | 1.1 |
| M120 | Grip strength | 1.1 | 1.1 | 1.1 |
| M120 | M120 | 4.7 | 4.7 | 4.7 |
| M120 | M120' | 4.3 | 4.3 | 4.3 |
| M120 | Risky alcohol use (yes) | 1.0 | 1.0 | 1.0 |
| M120 | Sedentary behaviour | 1.1 | 1.1 | 1.1 |
| M120 | Sleep duration | 1.1 | 1.0 | 1.1 |
| M120 | Smoking status (current) | 1.5 | 1.5 | 1.5 |
| M120 | Smoking status (former) | 1.4 | 1.4 | 1.4 |
| M120 | Socioeconomic status | 1.1 | 1.1 | 1.1 |
| M120 continuous | Body mass index | 1.2 | 1.2 | 1.2 |
| M120 continuous | CVD (yes) | 1.1 | 1.1 | 1.1 |
| M120 continuous | Diabetes (no) | 1.1 | 1.1 | 1.1 |
| M120 continuous | Employment (unemployed) | 1.1 | 1.1 | 1.1 |
| M120 continuous | Grip strength | 1.1 | 1.0 | 1.1 |
| M120 continuous | M120 continuous | 14.7 | 14.7 | 14.8 |
| M120 continuous | M120 continuous' | 14.5 | 14.5 | 14.6 |
| M120 continuous | Risky alcohol use (yes) | 1.0 | 1.0 | 1.0 |
| M120 continuous | Sedentary behaviour | 1.1 | 1.1 | 1.1 |
| M120 continuous | Sleep duration | 1.0 | 1.0 | 1.0 |
| M120 continuous | Smoking status (current) | 1.5 | 1.4 | 1.5 |
| M120 continuous | Smoking status (former) | 1.4 | 1.4 | 1.4 |
| M120 continuous | Socioeconomic status | 1.1 | 1.1 | 1.1 |
| M15 | Body mass index | 1.2 | 1.2 | 1.2 |
| M15 | CVD (yes) | 1.1 | 1.1 | 1.1 |
| M15 | Diabetes (no) | 1.1 | 1.1 | 1.1 |
| M15 | Employment (unemployed) | 1.1 | 1.1 | 1.1 |
| M15 | Grip strength | 1.1 | 1.1 | 1.1 |
| M15 | M15 | 3.7 | 3.7 | 3.7 |
| M15 | M15' | 3.2 | 3.2 | 3.2 |
| M15 | Risky alcohol use (yes) | 1.0 | 1.0 | 1.0 |
| M15 | Sedentary behaviour | 1.1 | 1.1 | 1.1 |
| M15 | Sleep duration | 1.0 | 1.0 | 1.1 |
| M15 | Smoking status (current) | 1.5 | 1.5 | 1.5 |
| M15 | Smoking status (former) | 1.4 | 1.4 | 1.4 |
| M15 | Socioeconomic status | 1.1 | 1.1 | 1.1 |
| M15 continuous | Body mass index | 1.2 | 1.2 | 1.2 |
| M15 continuous | CVD (yes) | 1.1 | 1.1 | 1.1 |
| M15 continuous | Diabetes (no) | 1.1 | 1.1 | 1.1 |
| M15 continuous | Employment (unemployed) | 1.1 | 1.1 | 1.1 |
| M15 continuous | Grip strength | 1.0 | 1.0 | 1.1 |
| M15 continuous | M15 continuous | 8.6 | 8.6 | 8.7 |
| M15 continuous | M15 continuous' | 8.3 | 8.3 | 8.3 |
| M15 continuous | Risky alcohol use (yes) | 1.0 | 1.0 | 1.0 |
| M15 continuous | Sedentary behaviour | 1.1 | 1.1 | 1.1 |
| M15 continuous | Sleep duration | 1.0 | 1.0 | 1.0 |
| M15 continuous | Smoking status (current) | 1.5 | 1.5 | 1.5 |
| M15 continuous | Smoking status (former) | 1.4 | 1.4 | 1.4 |
| M15 continuous | Socioeconomic status | 1.1 | 1.1 | 1.1 |
| M30 | Body mass index | 1.2 | 1.2 | 1.2 |
| M30 | CVD (yes) | 1.1 | 1.1 | 1.1 |
| M30 | Diabetes (no) | 1.1 | 1.1 | 1.1 |
| M30 | Employment (unemployed) | 1.1 | 1.1 | 1.1 |
| M30 | Grip strength | 1.1 | 1.1 | 1.1 |
| M30 | M30 | 4.2 | 4.1 | 4.2 |
| M30 | M30' | 3.7 | 3.7 | 3.7 |
| M30 | Risky alcohol use (yes) | 1.0 | 1.0 | 1.0 |
| M30 | Sedentary behaviour | 1.1 | 1.1 | 1.1 |
| M30 | Sleep duration | 1.0 | 1.0 | 1.1 |
| M30 | Smoking status (current) | 1.5 | 1.5 | 1.5 |
| M30 | Smoking status (former) | 1.4 | 1.4 | 1.4 |
| M30 | Socioeconomic status | 1.1 | 1.1 | 1.1 |
| M30 continuous | Body mass index | 1.2 | 1.2 | 1.2 |
| M30 continuous | CVD (yes) | 1.1 | 1.1 | 1.1 |
| M30 continuous | Diabetes (no) | 1.1 | 1.1 | 1.1 |
| M30 continuous | Employment (unemployed) | 1.1 | 1.1 | 1.1 |
| M30 continuous | Grip strength | 1.1 | 1.0 | 1.1 |
| M30 continuous | M30 continuous | 14.0 | 14.0 | 14.0 |
| M30 continuous | M30 continuous' | 13.6 | 13.6 | 13.6 |
| M30 continuous | Risky alcohol use (yes) | 1.0 | 1.0 | 1.0 |
| M30 continuous | Sedentary behaviour | 1.1 | 1.1 | 1.1 |
| M30 continuous | Sleep duration | 1.0 | 1.0 | 1.0 |
| M30 continuous | Smoking status (current) | 1.5 | 1.5 | 1.5 |
| M30 continuous | Smoking status (former) | 1.4 | 1.4 | 1.4 |
| M30 continuous | Socioeconomic status | 1.1 | 1.1 | 1.1 |
| M5 | Body mass index | 1.2 | 1.2 | 1.2 |
| M5 | CVD (yes) | 1.1 | 1.1 | 1.1 |
| M5 | Diabetes (no) | 1.1 | 1.1 | 1.1 |
| M5 | Employment (unemployed) | 1.1 | 1.1 | 1.1 |
| M5 | Grip strength | 1.1 | 1.1 | 1.1 |
| M5 | M5 | 3.5 | 3.5 | 3.5 |
| M5 | M5' | 3.1 | 3.1 | 3.1 |
| M5 | Risky alcohol use (yes) | 1.0 | 1.0 | 1.0 |
| M5 | Sedentary behaviour | 1.1 | 1.1 | 1.1 |
| M5 | Sleep duration | 1.0 | 1.0 | 1.1 |
| M5 | Smoking status (current) | 1.5 | 1.5 | 1.5 |
| M5 | Smoking status (former) | 1.4 | 1.4 | 1.4 |
| M5 | Socioeconomic status | 1.1 | 1.1 | 1.1 |
| M5 continuous | Body mass index | 1.2 | 1.2 | 1.2 |
| M5 continuous | CVD (yes) | 1.1 | 1.1 | 1.1 |
| M5 continuous | Diabetes (no) | 1.1 | 1.1 | 1.1 |
| M5 continuous | Employment (unemployed) | 1.1 | 1.1 | 1.1 |
| M5 continuous | Grip strength | 1.0 | 1.0 | 1.1 |
| M5 continuous | M5 continuous | 5.0 | 5.0 | 5.0 |
| M5 continuous | M5 continuous' | 4.7 | 4.7 | 4.8 |
| M5 continuous | Risky alcohol use (yes) | 1.0 | 1.0 | 1.0 |
| M5 continuous | Sedentary behaviour | 1.1 | 1.1 | 1.1 |
| M5 continuous | Sleep duration | 1.0 | 1.0 | 1.0 |
| M5 continuous | Smoking status (current) | 1.5 | 1.5 | 1.5 |
| M5 continuous | Smoking status (former) | 1.4 | 1.4 | 1.4 |
| M5 continuous | Socioeconomic status | 1.1 | 1.1 | 1.1 |
| M60 | Body mass index | 1.2 | 1.2 | 1.2 |
| M60 | CVD (yes) | 1.1 | 1.1 | 1.1 |
| M60 | Diabetes (no) | 1.1 | 1.1 | 1.1 |
| M60 | Employment (unemployed) | 1.1 | 1.1 | 1.1 |
| M60 | Grip strength | 1.1 | 1.1 | 1.1 |
| M60 | M60 | 4.7 | 4.7 | 4.7 |
| M60 | M60' | 4.3 | 4.3 | 4.3 |
| M60 | Risky alcohol use (yes) | 1.0 | 1.0 | 1.0 |
| M60 | Sedentary behaviour | 1.1 | 1.1 | 1.1 |
| M60 | Sleep duration | 1.0 | 1.0 | 1.1 |
| M60 | Smoking status (current) | 1.5 | 1.5 | 1.5 |
| M60 | Smoking status (former) | 1.4 | 1.4 | 1.4 |
| M60 | Socioeconomic status | 1.1 | 1.1 | 1.1 |
| M60 continuous | Body mass index | 1.2 | 1.2 | 1.2 |
| M60 continuous | CVD (yes) | 1.1 | 1.1 | 1.1 |
| M60 continuous | Diabetes (no) | 1.1 | 1.1 | 1.1 |
| M60 continuous | Employment (unemployed) | 1.1 | 1.1 | 1.1 |
| M60 continuous | Grip strength | 1.1 | 1.0 | 1.1 |
| M60 continuous | M60 continuous | 29.8 | 29.7 | 29.8 |
| M60 continuous | M60 continuous' | 29.3 | 29.2 | 29.4 |
| M60 continuous | Risky alcohol use (yes) | 1.0 | 1.0 | 1.0 |
| M60 continuous | Sedentary behaviour | 1.1 | 1.1 | 1.1 |
| M60 continuous | Sleep duration | 1.0 | 1.0 | 1.0 |
| M60 continuous | Smoking status (current) | 1.5 | 1.5 | 1.5 |
| M60 continuous | Smoking status (former) | 1.4 | 1.4 | 1.4 |
| M60 continuous | Socioeconomic status | 1.1 | 1.1 | 1.1 |
| Physical activity duration | Body mass index | 1.2 | 1.2 | 1.2 |
| Physical activity duration | CVD (yes) | 1.1 | 1.1 | 1.1 |
| Physical activity duration | Diabetes (no) | 1.1 | 1.1 | 1.1 |
| Physical activity duration | Employment (unemployed) | 1.1 | 1.1 | 1.1 |
| Physical activity duration | Grip strength | 1.1 | 1.1 | 1.1 |
| Physical activity duration | Physical activity duration | 4.0 | 4.0 | 4.0 |
| Physical activity duration | Physical activity duration' | 3.7 | 3.7 | 3.7 |
| Physical activity duration | Risky alcohol use (yes) | 1.0 | 1.0 | 1.0 |
| Physical activity duration | Sedentary behaviour | 1.1 | 1.1 | 1.1 |
| Physical activity duration | Sleep duration | 1.1 | 1.1 | 1.1 |
| Physical activity duration | Smoking status (current) | 1.5 | 1.5 | 1.5 |
| Physical activity duration | Smoking status (former) | 1.4 | 1.4 | 1.4 |
| Physical activity duration | Socioeconomic status | 1.1 | 1.1 | 1.1 |
| Physical activity volume | Body mass index | 1.2 | 1.2 | 1.2 |
| Physical activity volume | CVD (yes) | 1.1 | 1.1 | 1.1 |
| Physical activity volume | Diabetes (no) | 1.1 | 1.1 | 1.1 |
| Physical activity volume | Employment (unemployed) | 1.1 | 1.1 | 1.1 |
| Physical activity volume | Grip strength | 1.1 | 1.1 | 1.1 |
| Physical activity volume | Physical activity volume | 3.9 | 3.9 | 3.9 |
| Physical activity volume | Physical activity volume' | 3.4 | 3.4 | 3.4 |
| Physical activity volume | Risky alcohol use (yes) | 1.0 | 1.0 | 1.0 |
| Physical activity volume | Sedentary behaviour | 1.1 | 1.1 | 1.1 |
| Physical activity volume | Sleep duration | 1.1 | 1.0 | 1.1 |
| Physical activity volume | Smoking status (current) | 1.5 | 1.5 | 1.5 |
| Physical activity volume | Smoking status (former) | 1.4 | 1.4 | 1.4 |
| Physical activity volume | Socioeconomic status | 1.1 | 1.1 | 1.1 |
| *VIFs were calculated separately for each imputed dataset and summarized as the mean and range across imputations.* | | | | |
